# *Cherax warsamsonicus*, a new species of crayfish from the Kepala Burung (Vogelkop) peninsula in West Papua, Indonesia (Crustacea, Decapoda, Parastacidae)

**DOI:** 10.3897/zookeys.660.11847

**Published:** 2017-03-13

**Authors:** Christian Lukhaup, Rury Eprilurahman, Thomas von Rintelen

**Affiliations:** 1 Waldstrasse 5a, 66999 Hinterweidenthal, Germany; 2 Animal Systematics Laboratory, Faculty of Biology, Universitas Gadjah Mada Jl. Teknika Selatan, Sekip Utara Yogyakarta 55281, Indonesia; 3 Museum für Naturkunde -Leibniz Institute for Evolution and Biodiversity Science, Invalidenstraße 43, 10115 Berlin, Germany

**Keywords:** *Cherax*, Crustacea, Decapoda, morphology, New Guinea, Parastacidae, pet trade, taxonomy, Warsamson River

## Abstract

A new species, *Cherax warsamsonicus* **sp. n.**, endemic to the Warsamson River drainage, in the western part of the Kepala Burung (Vogelkop) peninsula, West Papua, Indonesia, is described, figured and compared with its closely related species, *Cherax misolicus* Holthuis, 1949. The new species may be easily distinguished from *C. misolicus* by the shape of the rostrum, absence of setae on the rostrum, the shape of the chelae, the presence of 3-4 cervical spines and by using sequence divergence, which is substantial for considering *C. warsamsonicus* **sp. n.** to be a new species. The new species is collected and exported for ornamental purposes and its commercial name in the pet trade is *Cherax* “irian jaya”, *Cherax* “pink coral”, or *Cherax* “hoa creek“. Due to similar colouration it is often confused with the recently described *Cherax pulcher* Lukhaup, 2015.

## Introduction

The crayfishes of the island of New Guinea were extensively studied by Holthuis (1949, 1956, 1958, 1982, 1986, 1996), with additions by Lukhaup and Pekny (2006, 2008a), Lukhaup and Herbert (2008), Lukhaup (2015), Lukhaup et al. (2015) and Patoka et al. (2015). Nevertheless, over the last decade, there has been an increasing number of colourful crayfish, presumed to be a further undescribed species, sold from New Guinea in the ornamental fish trade in Europe and Asia under the names *Cherax* “irian jaya” and *Cherax* “hoa creek” (Lukhaup and Pekny 2014). These have been exported to some countries in Europe, East Asia and North America. While they are clearly species of *Cherax*, a large genus of freshwater crayfish occurring in Indonesia (West Papua), Papua New Guinea and Australia, their exact provenances could not be ascertained, with dealers claiming they came from Ajamaru (West Papua) and other places in the area that could not be confirmed. Also species have been mixed at the places of exporters in Sorong and Jakarta. Therefore in January 2016 we visited the Sorong Regency and South Sorong Regency to clarify the distribution of some of the species present in the pet trade. In the present contribution, this species is described as new to science and establish that it is in fact native to the Warsamson River Drainage, Sorong Regency of the Kepala Burung (Vogelkop) Peninsula West Papua, Indonesia. *Cherax warsamsonicus* sp. n., is genetically and morphologically most similar to *Cherax misolicus* Holthuis, 1949 endemic to the Island of Misool, one of four major islands in the Raja Ampat Islands in West Papua, Indonesia and two other undescribed species from Sorong and South Sorong Recency.

*Cherax misolicus* and *Cherax warsamsonicus* sp. n. may be easily distinguished using sequence divergence, by colouration and pattern of live individuals, by the shape of the chelae, the shape of rostrum, and presence of dense setae on the rostrum in *C. misolicus* which is absent in the new species.

## Materials and methods

Samples of *Cherax warsamsonicus* sp. n. as well as three other species were collected from streams in the southwestern part of the Kepala Burung peninsula in February 2016. In addition, sequence from seven species of *Cherax* and from two other parastacid genera used as outgroup were downloaded from GenBank (see Table 1). Holotype and allotype were photographed and kept alive in indoor tanks until samples were obtained for DNA analysis. After this procedure animas were preserved in 70 % ethanol. Morphometric parameters of all individuals were taken using an electronic digital calliper with an accuracy of 0.1 mm.

**Table 1.** Material studied with GenBank accession numbers.

Species/sample	Location	GenBank acc. nos		Source
		COI	16S	
*Cherax albertisii*	Bensbach River, Papua New Guinea (Queensland Museum)	–	KJ920770	Eprilurahman et al., unpubl.
*C. boesemani*	Ajamaru Lake, Papua Barat; 1°17'19.97"S, 132°14'49.14"E; January 23, 2016	#	#	this study
		#	#	
*C. holthuisi*	Papua Barat	KU821419	KU821433	Blaha et al. 2016
*C. misolicus*	Misool Island, South of Papua Barat (Leiden Museum)	–	KJ920813	Eprilurahman et al., unpubl.
*C. monticola*	Baliem River, Wamena, Papua	KF649851	KF649851	Gan et al. 2014
		–	KJ920818	
*C. paniaicus*	Lake Tage, Papua (Field collection)	KJ950528	KJ920830	Eprilurahman et al., unpubl.
*C. peknyi*	Pet Shop	KU821422	KU821435	Blaha et al. 2016
*C. pulcher*	Hoa Creek (Teminabuan), Papua Barat; 1°28'32.73"S 132° 3'54.94"E; January 23, 2016	#	#	this study
*C. pulcher*‘	Papua Barat (Pet Shop)	KU821424	KU821438	Blaha et al. 2016
		KU821426	KU821437	Blaha et al. 2016
*C. rhynchotus*	Lake Wicheura, Cape York, Queensland (Queensland Museum)	–	KJ920765	Eprilurahman et al., unpubl.
*C. snowden*	Oinsok (Ainsok River Drainage), Papua Barat; 1°11'40.07"S 131°50'1.14"E; January 24, 2016	#	#	this study
*C. warsamsonicus*	Small tributary to Warsamson River Collection Date : January 20 ,2016 0°49'16.62"S, 131°23'3.34"E	#	#	this study
*Engaeus strictifrons*	Crawford River, Victoria, Australia	AF493633	AF492812	Munasinghe et al. 2003
*Euastacus bispinosus*	Crawford River, Victoria, Australia	AF493634	AF492813	Munasinghe et al. 2003

# = No. pending, will be entered during revision process.

DNA was purified from 2 mm³ of muscle tissue with a Qiagen BioSprint 96 using the manufacturer’s protocol. Polymerase chain reaction (PCR) was used to amplify two mitochondrial gene fragments, a ~535 bp region of the 16S ribosomal RNA gene (16S) using primers 1471 and 1472 (Crandall and Fitzpatrick 1996) and a 710 bp fragment of the Cytochrome Oxidase subunit I gene (COI) using primers LCO1490 and HCO2198 (Folmer et al. 1994).

PCR was performed in 25 µl volumes containing 1x Taq buffer, 1.5 mM MgCl2, 200 µM each dNTP, 1 U Taq polymerase, ca. 50-100 ng DNA and ddH2O. After an initial denaturation step of 3 min at 94 °C, cycling conditions were 35 cycles at 94 °C for 35 s, 45 °C (COI) or 50 °C (16S) for 60 s, and 72 °C for 1 min (COI) or 90 s (16S), with a final elongation step of 5 min at 72 °C. The same primers were used in PCR and sequencing. PCR products were sent to Macrogen Europe for purification and cycle sequencing of both strands of each gene.

Sequences were aligned by eye (COI) and with MAFFT (16S) using the G-INS-i strategy suitable for thorough alignments of sequences with global homology (Katho et al. 2002). The resulting alignments had a length of 658 bp (COI) and 543 bp (16S), respectively. To determine the best substitution model for Bayesian inference analyses (see below), hierarchical likelihood ratio tests were carried out with jModelTest (Posada 2008) on both sequence sets (24 models tested). Based on the Akaike Information Criterion and the Bayesian Inference Criterion, the GTR + I + G (COI) and the GTR + G (16S) models were chosen. The two sequence datasets were subsequently analysed both separately and combined.

Phylogenetic trees were reconstructed by maximum parsimony (MP) using the heuristic search algorithm as implemented in PAUP* (Swofford 2002), with gaps treated as fifth base. Support for nodes was estimated by bootstrap analysis (1,000 bootstrap replicates with 10 random addition sequence replicates each). Maximum Likelihood (ML) analyses were conducted with RAxML (Stamatakis et al. 2008; RAxML BlackBox; 100 bootstrap replicates) under the GTR + (I) + G model of sequence evolution. In addition, Bayesian inference (BI) was employed to infer phylogeny by using MrBayes 3.2.2 (Ronquist and Huelsenbeck 2003). The MCMCMC-algorithm was run with four independent chains for 5,000,000 generations, samplefreq = 250, and burnin = 10,001) using the models specified above.

The combined dataset was subjected to a partitioned analysis (ML and BI) using the different models for the two genes in the BI analyses. All new sequences have been deposited in GenBank, see Table 1).

## Systematics

### Parastacidae Huxley, 1879

#### Genus *Cherax* Erichson, 1846

##### Cherax warsamsonicus sp. n.

Taxon classificationAnimaliaORDOFAMILIA

http://zoobank.org/4A8CC447-7082-4105-A676-BDB4B6092D95

**Figure 1.**
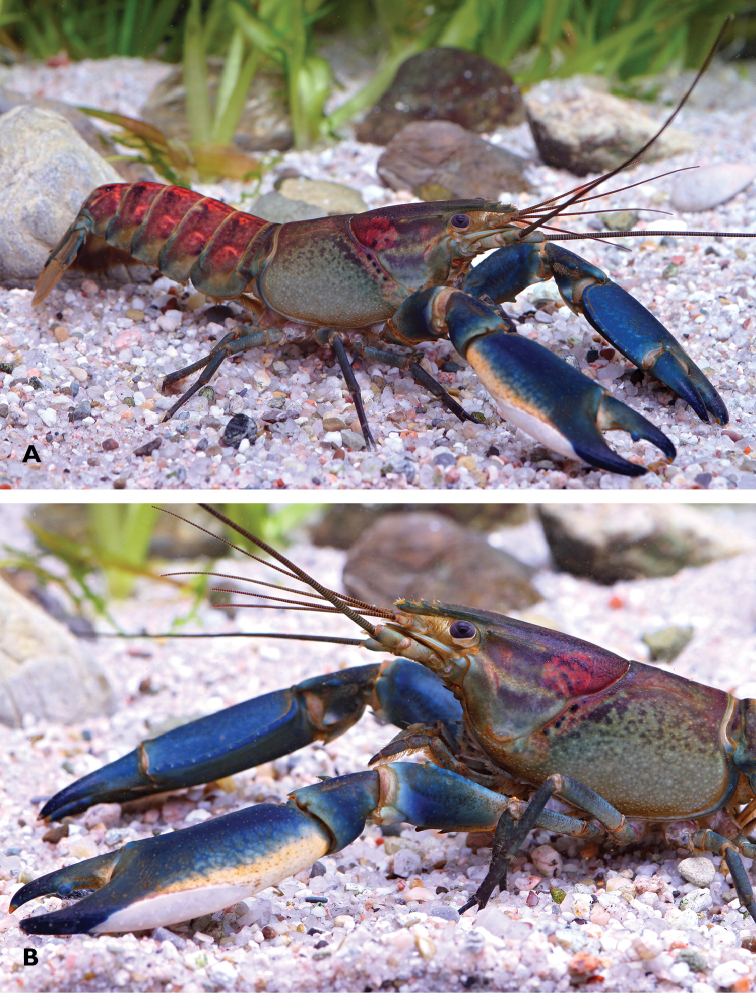
*Cherax warsamsonicus* sp. n. **A** holotype male (MZB Cru 4529) from the Warsamson River, South Sorong Regency **B** idem, side view.

**Figure 2.**
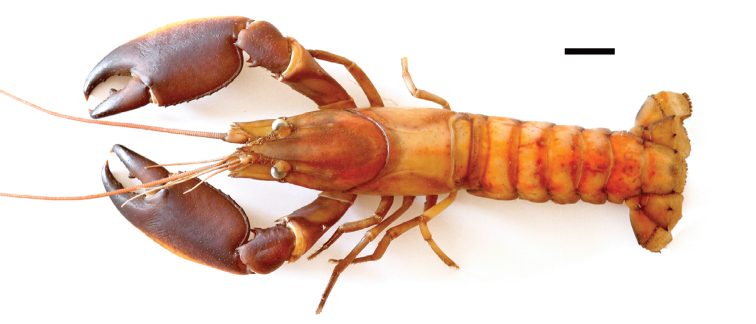
*Cherax warsamsonicus* sp. n. holotype male (MZB Cru 4529). Scale bar: 10 mm.

**Figure 3.**
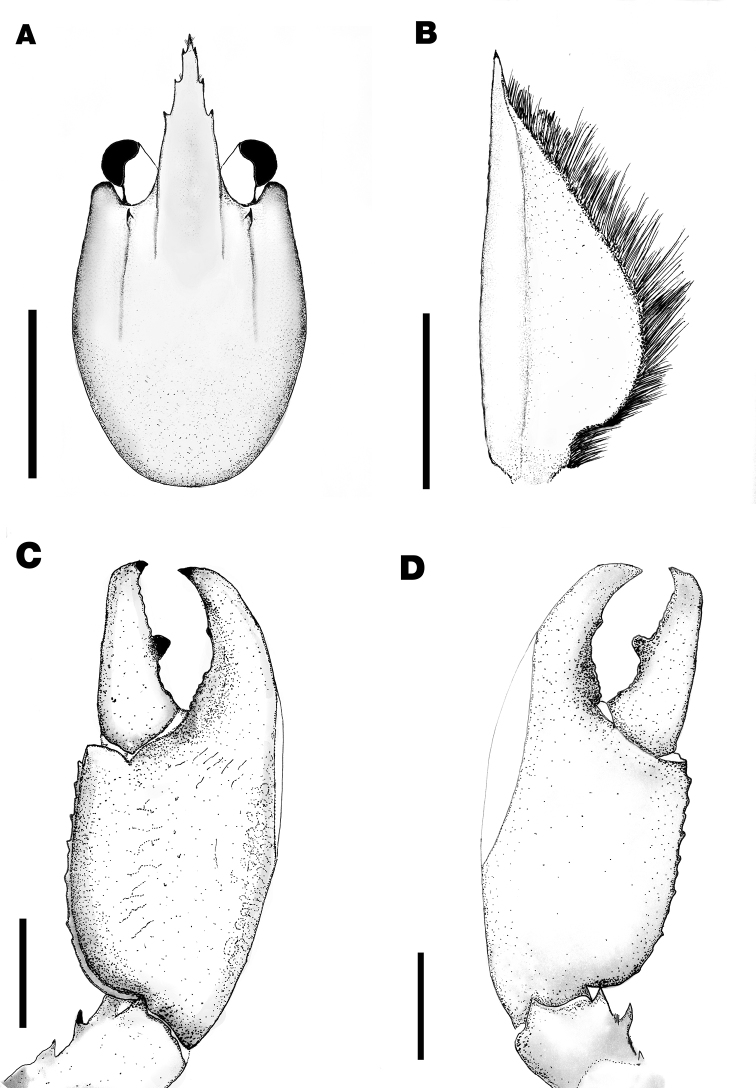
*Cherax warsamsonicus* sp. n. holotype male (MZB Cru 4529). **A** dorsal view of carapace **B** scaphocerite **C** dorsal view of right chelae **D** ventral view of left chelae. Scale bars: **A, C, D** 10 mm, **B** 5mm.

**Figure 4.**
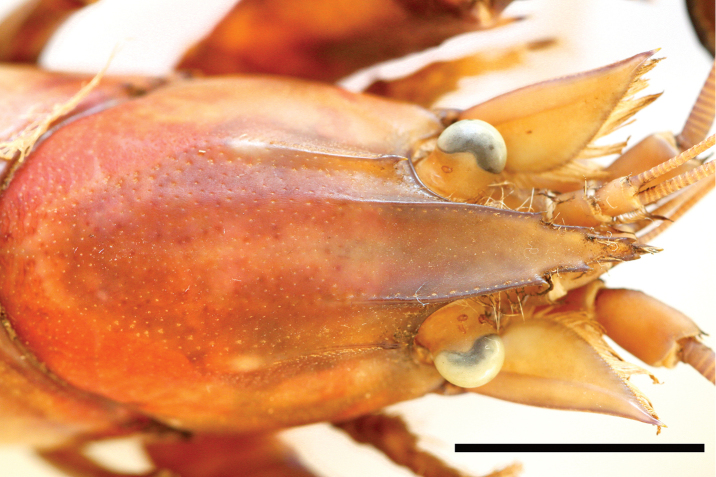
*Cherax warsamsonicus* sp. n. holotype male (MZB Cru 4529), dorsal view of cephalothorax. Scale bar: 10 mm.

**Figure 5.**
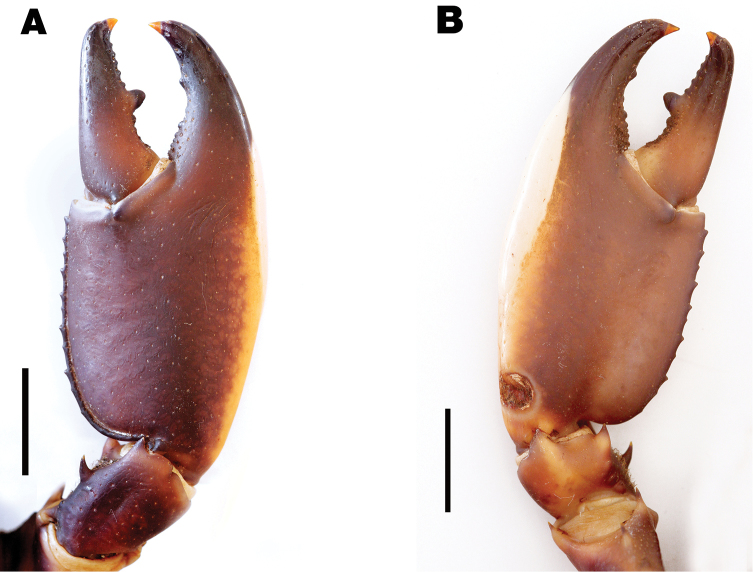
*Cherax warsamsonicus* sp. n. holotype male (MZB Cru 4529). **A** right first chela, dorsal aspect **B** right first chela, ventral aspect. Scale bars: 10 mm.

Figs 1 , 2 , 3 , 4 , 5

###### Material examined.

**Holotype**: male (MZB Cru 4529), among roots along banks of a unnamed creek draining into Warsamson River, north of Sorong City, 0°49'16.62"S 131°23'3.34"E, West Papua, Indonesia. coll. Chris Lukhaup, Irianto Wahid and unnamed local guide January 20 2016. **Allotype**: female (MZB Cru 4530), same data as holotype. **Paratypes**: (MZB Cru 4531), same data as holotype.

###### Diagnosis.

Carapace surface smooth with four small spiniform tubercles posterior to cervical groove on lateral carapace. Eyes large, pigmented. Cornea slightly broader than eyestalk. Rostrum lanceolate in shape with excavated margins. Rostral margins with three prominent teeth. Rostral carinae prominent. Postorbital ridges prominent with one acute tubercle at anterior terminus. Uncalcified patch on lateral margin of chelae of adult male white, translucent. Propodal cutting edge with row of small granules and one large tubercle. Chelipeds blue and white with white joints. Fingers blue in distal third black with hooked tips. Other walking legs blue-gray. Pleon black with pinkish-red pattern. Lateral pleura lighter becoming greyish green.

###### Description of male holotype

**(Figs 2–5).** *Body* and eyes pigmented. Eyes not reduced. Body subovate, slightly compressed laterally. Pleon narrower then cephalothorax (width 16.7 mm and 17.5 mm respectively). Rostrum (Fig. 3A) broad in shape, reaching nearly to end of ultimate antennular peduncle and one third longer than wide (width 5 mm at base, length 13.6 mm). Upper surface smooth, slightly scattered. Margins slightly elevated continuing in rostral carinae on carapace, almost straight in basal part, distally rather moderately tapering towards apex. Lateral rostral margin bearing three prominent teeth in distal half, pointing upwards at angle of approximately 45°. Few short hairs present on distal half of outer margins. Acumen with anteriorly orientated spine.

*Rostral carinae* extending as slight elevation posteriorly on carapace terminating at ending of postorbital ridges. Postorbital ridges well developed, terminating in spiniform tubercle anteriorly, fading at two-thirds of occipital carapace length, posteriorly. Dorsal surface of carapace smooth, slightly pitted, cervical and branchiocardiac grooves distinct, non-setose, one prominent corneous spine and three tubercles present at middle part behind cervical groove on lateral sides of carapace.

*Areola* length 13.7 mm, narrowest width 7.4 mm. Length of areola 31.8% of total length of carapace (43 mm).

Ventrolateral parts smooth with scattered pits; anterior margin strongly produced, rounded upper margin directed inward.

*Scaphocerite* (Fig. 3B) broadest at midlength, convex in distal part becoming narrower in basal part; thickened lateral margin terminating in large corneous spine, almost reaching distal margin of ultimate segment of antennular peduncle. Right scaphocerite 11 mm long and 4 mm wide. Proximal margins setose. Antennulae and antennae typical for genus. Antennae similarly long as body. Antennular peduncle reaching slightly behind acumen, antennal peduncle reaching slightly behind apex of scaphocerite. Antennal protopodite with spine anteriorly; basicerite with one lateral and one ventral spine.

*Mouthparts* typical for the genus. Epistome with subcordiform cephalic lobe anteriorly bearing lanceolate cephalomedian projection constricted at base. Lateral margins of lobe not thickened; each lateral margin with two groups of 8-9 tubercles separated by a smooth place. Central part smooth, not pitted, excavate. Eyes rather large; cornea globular, darkly pigmented, nearly as long as eyestalk; eyestalk slightly narrower than cornea.

*First pereopod* equal in form, chela slightly gaping, equal in size, right cheliped (39 mm long, 8.2 mm high, 16.5 mm wide). Left chelae (Fig. 3C–D) 38.3 mm long and 8.2 mm high, 16.5 mm wide, strongly compressed. Fingers shorter than palm (dactylus 15.3 mm long). Dactylus broad at base (7 mm), tapering slightly towards tip.

Tip with sharp, corneous, hooked tooth pointing outwards at an angle of 45°. Cutting edge of dactyl with continuous row of rather small granular teeth and one prominent larger tooth at middle of cutting edge. Ventral and dorsal surface of movable finger with scattered punctuation. Posterior half of cutting edge with slightly rounded gap. Fixed finger triangular, merging gradually into palm, ending in sharp, corneous, hooked tooth, standing almost perpendicular to axis of finger. Tips of fingers slightly crossing when fingers clasp. Upper surface of palm practically smooth, slightly pitted, more densely pitted at margins. Fixed finger with approximately same width as dactyl at base (7.3 mm). Few scattered short setae present in posterior ventral part of fixed finger. Cutting edge of fixed finger with row of rather small granular teeth at posterior half and one at middle of anterior part.

Dorsal surface of carpus (11.77 mm) smooth and pitted, with slight excavation in middle part and with well-developed acute and hooked spiniform tubercle in middle of dorsolateral inner margin. Ventral carpal surface margins slightly elevated, non-setose and with fovea; inner margin with one acute spiniform tubercle oriented in angle of approx. 45°; outer margin smooth with one spiniform tubercle oriented almost anteriorly.

*Merus* (19.2 mm) laterally depressed in basal part; surface slightly pitted; one prominent spine at anterior part at dorsal surface. Row of 12-13 small granules on inner ventrolateral margin, four prominent spines, one at midlength other in middle of anterior part, third on distal ventrolateral outer margin, fourth on distal ventrolateral inner margin.

Ischium (10.8 mm) smooth with small spine and three granules at midlength of ventrolateral inner margin.

*Second pereopod* reaching anteriorly to approximately middle of scaphocerite. Finger as long as palm (5.6 mm), of same height. Short setae present on dactyl and fixed finger, getting denser anteriorly. Cutting edge of fixed finger and carpus with row of short setae. Carpus, smooth, not pitted, slightly longer than palm. Merus (12.7 mm) 1.7 times longer than carpus (7.2 mm). Ischium (6.2 mm) half as long as merus.

*Third pereopod* overreaching second by almost length of finger of second pereopods. Fingers shorter than palm.Fourth pereopod reaching distal margin of scaphocerite. Dactylus with corneous tip. Short scattered setae present. Propodus more than twice as long as dactylus, nearly 1.5 times as long as carpus; somewhat flattened, carrying many stiff setae on lower margin. Merus just slightly longer than propodus.

*Fifth pereopod* similar to fourth, slightly shorter.

Dorsal surface of pleon smooth, with scattered pits; abdominal segments with short setae present on caudal margins.

*Telson* with posterolateral spines, dense short setae present in posterior third. Posterior margins setose. Uropodal protopod with distal spine on mesial lobe. Exopod of uropod with transverse row of posteriorly directed diminutive spines ending in one more prominent spine, posteriorly directed on outer margin of mesial lobe. Terminal half of exopod with small tubercles and short hairs, slightly corrugated. Endopod of uropod smooth. Short scattered hairs present on posterior third of dorsal exopod. Postrolateral spine on outer margin present. Second spine on medial dorsal surface present, directed posteriorly.

###### Description of allotype female

**Figure 6.**
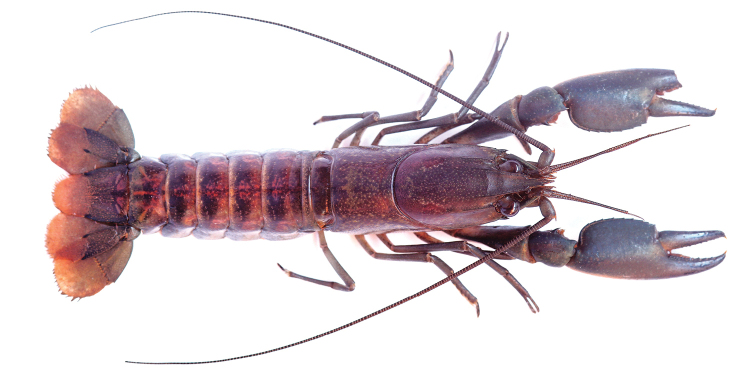
*Cherax warsamsonicus* sp. n., allotype female (MZB Cru 4530).

(Fig. 6). Chela of first pereopods equal, 2.5 times as long as broad (24.5 mm and 9 mm respectively). Mesial margin of palm slightly elevated, forming slender serrated ridge with row of 9 small granular teeth. Cutting edge of dactylus with 8-9 rather small granular teeth. Cutting edge of fixed finger with 8-9 small granules. Small scattered short setae visible along ventral cutting edge of chelae, more dense and long in ventral posterior area. Tips of fingers slightly crossing when fingers clasp, not gaping. Cervical groove distinct, non-setose. Pleon just slightly narrower than cephalothorax (widths 12 mm and 12.5 mm respectively). Same colour pattern as in males, less intense.

*Size.* The biggest male examined has a carapace length of 48.7 mm, and a total length of 109 mm (n = 4) ,the holotype male has a total length of 92,8 mm the other males have a total length of 73mm and 96 mm; the female has a carapace length of 31.8 mm and a total length of 73 mm (n = 1).

*Colour.* The living animals (Fig. 1A, B) are coloured as follows. Male: Chelae dark blue with white margins and white patch. Anterior part usually dark blue. Corneous tooth on tip of fingers orange. Cephalothorax greenish black, with small slightly darker spots laterally, fading ventrally to grey-green. Pink to pinkish red patch on dorsolateral side of the carapace between rostral carinae and cervical grove. Segments of pleon with pinkish red band anteriorly becoming black in posterior part. Lateral pleura slightly lighter becoming greyish green. Walking legs blue to dark bluish grey. Distal margin of tail-fan creamy orange to orange. Females: usually greyish green to bluish grey with bluish chelae and a white margin.

###### Molecular phylogenetic results.

**Figure 7.**
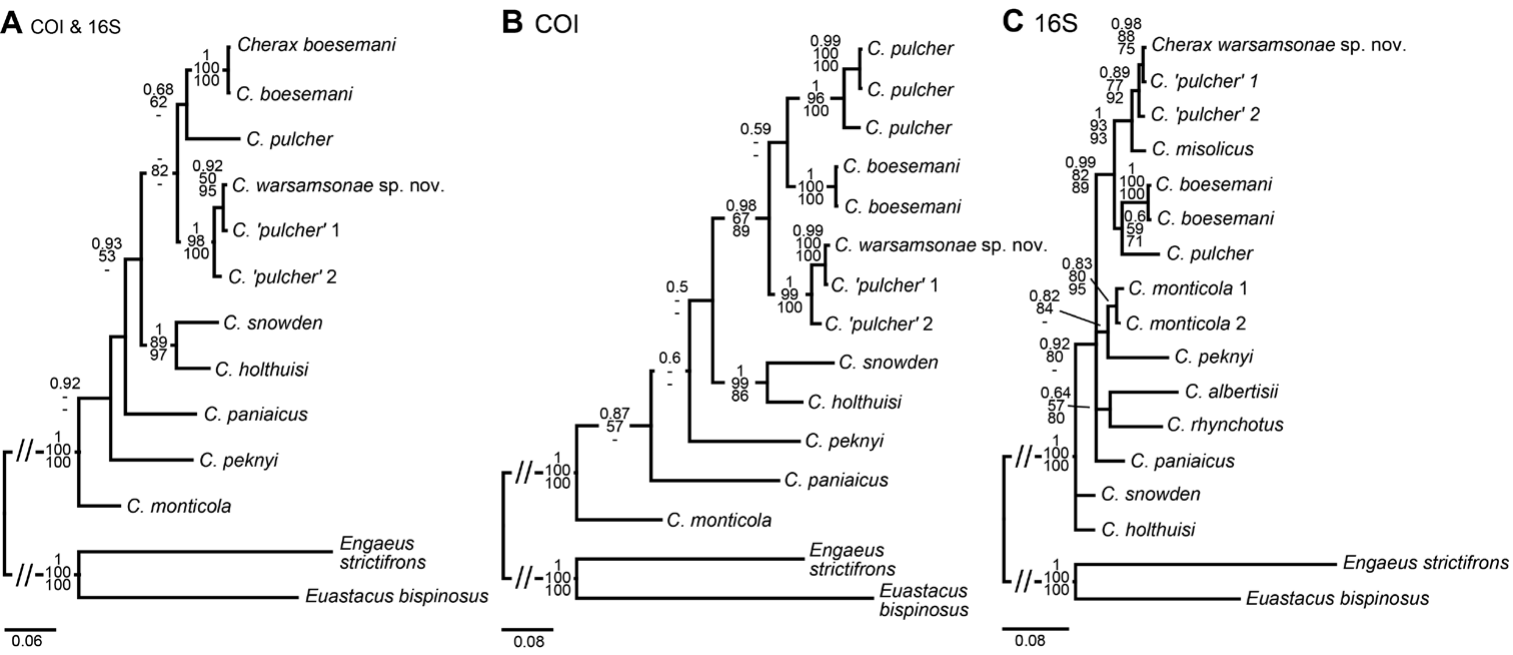
Phylogenetic position of *Cherax warsamsonicus* sp. n. within closely related New Guinean *Cherax* species, reconstructed by BI analyses of two mitochondrial gene fragments. Number on branches show, from top, Bayesian posterior probabilities and ML/MP bootstrap values. The scale bar indicates the substitution rate. See Table 1 for information on the sequenced specimens. **A** Topology based on concatenated COI and 16S dataset **B** Topology based on COI dataset **C** Topology based on 16S dataset.

*Cherax warsamsonicus* sp. n. clusters with two sequences retrieved from GenBank as *C. pulcher* and the entire cluster forms a well-supported clade with *Cherax misolicus* (16S only, Fig. 7C). The *C. ‘pulcher*’ sequences from GenBank almost certainly belong to *C. warsamsonicus* sp. n., as the included verified sequence of *C. pulcher* from a topotypical specimen is shown to be quite distinct and one of the two GenBank derived sequences is identical to the *C. warsamsonicus* sequence generated in this study. *C. warsamsonicus* sp. n. is well isolated from *C. misolicus* with a sequence divergence (p-distance, 16S) of 1.9-2.1 %, respectively, supporting the morphology-based description of *C. warsamsonicus* as a new species.

###### Deposition of types.

The holotype (MZB Cru 4529), allotype (MZB Cru 4530) and paratypes (MZB Cru 4531) are deposited at the Museum Zoologicum Bogoriense (= Bidang Zoologi) Reseach Centre for Biology (=Pusat Penelitian Biologi), Indonesian Institute of Sciences (= LIPI), Jalan Raya Jakarta-Bogor Km 46 Cibinong 16911, Indonesia.

###### Systematic position.

Holthuis (1949) in his publication on the New Guinea *Cherax* considered species should be placed into two groups. One with the rostral and median carinae absent or weakly developed and referred to as the *Cherax* group following the characteristics of the type species, *C. preissii* (Erichson) from southwest Australia. The other group contains species that have the rostral and sometimes the median carina well developed and referred to as the *Astaconephrops* group with Nobili’s (1899) *Astaconephrops albertisii* as the type. Newly described species have been placed into one or the other of the two subgenera (Lukhaup and Pekny 2006; Lukhaup and Pekny 2008; Lukhaup and Herbert 2008; Lukhaup 2015, Lukhaup et al. 2015; Patoka, Blaha and Kouba 2015). Munasinghe et al. (2004a, b), Austin (1996); and Austin et al. (1996) however, identified three geographically-based lineages within *Cherax* based on molecular genetics and phylogenetic studies. These consist of a southwestern group, an eastern group and a northern group. Support for the latter group however was based on only very limited sampling (e.g. single samples of *C. quadricarinatus*, *C. rhynchotus* and *C. peknyi* in Munasinghe et al. study). Munasinghe et al. (2004b) indicate that the division of *Cherax* into two subgenera, as conceived by Holthuis and subsequent authors dealing with New Guinea crayfish has to be reconsidered. Based on Munasinghe et al. (2004), Austin (1996), and Austin et al. (1996a). *Cherax warsamsonicus* sp. n. belongs to the northern species group lineage consisting of 22 species:

*C. albertisii* (Nobili, 1899)

*C. boesemani* Lukhaup & Pekny, 2008

*C. boschmai* Holthuis, 1949

*C. buitendijkae* Holthuis, 1949

*C. communis* Holthuis, 1949

*C. divergens* Holthuis, 1950

*C. gherardii* Patoka, Bláha & Kouba, 2015

*C. holthuisi* Lukhaup & Pekny, 2006

*C. lorentzi aruanus* (Roux, 1911)

*C. lorentzi lorentzi* (Roux, 1911)

*C. longipes* Holthuis, 1949

*C. misolicus* Holthuis, 1949

*C. murido* Holthuis, 1949

*C. monticola* Holthuis, 1950

*C. minor* Holthuis, 1996

*C. peknyi* Lukhaup & Herbert, 2008

*C. pallidus* Holthuis, 1949

*C. papuanus* Holthuis, 1949

*C. paniaicus* Holthuis, 1949

*C. pulcher* Lukhaup, 2015

*C. solus* Holthuis, 1949

*C. snowden* Lukhaup, Panteleit & Schrimpf, 2015

In comparison to all species of the northern group the new species, *C. warsamsonicus*, is most similar to *C. misolicus*, a species that is endemic to Misool Island, one of four major islands in the Raja Ampat Islands in West Papua, Indonesia.

**Figure 8.**
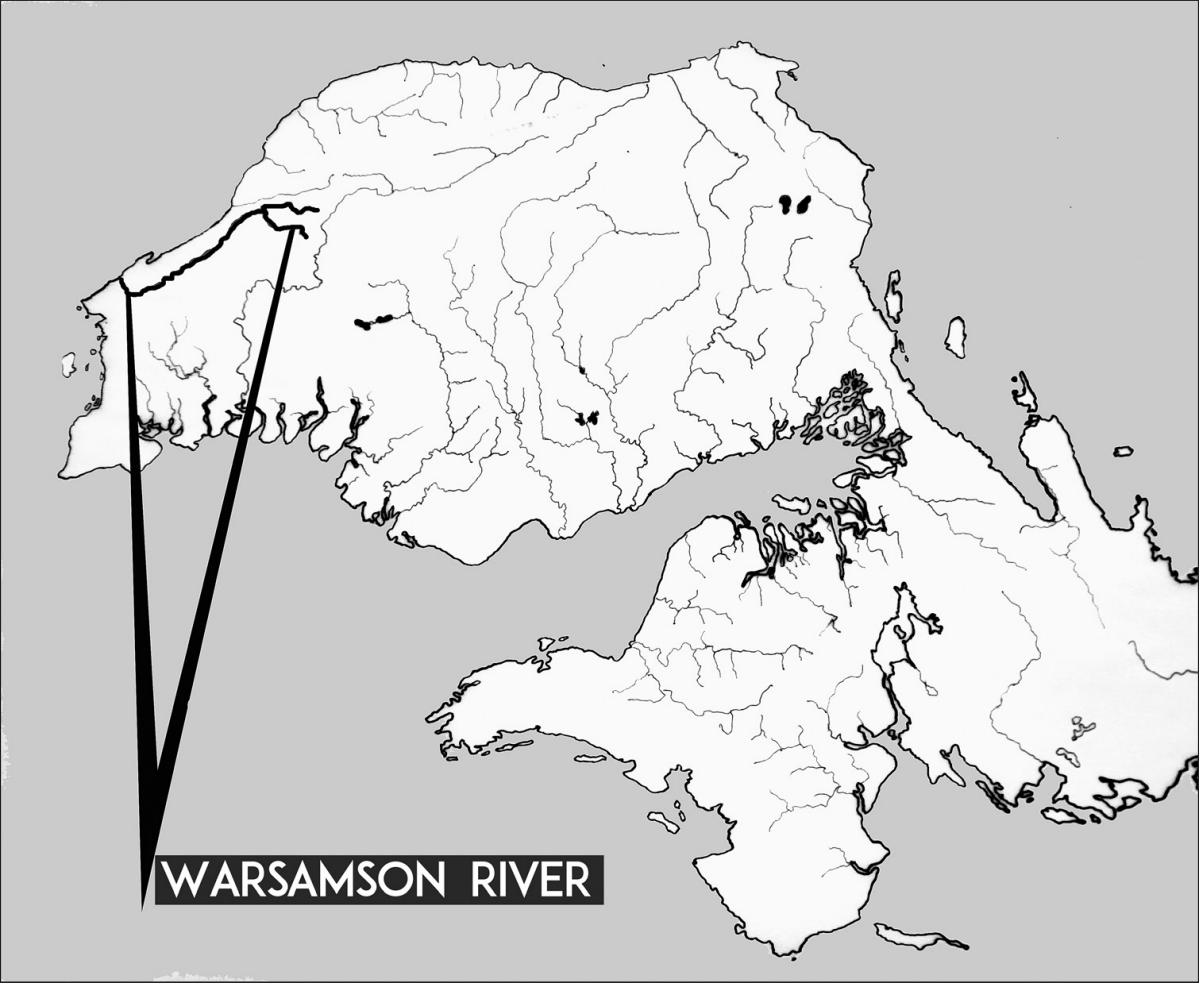
Rostrum dorsal view **A** *Cherax warsamsonicus* sp. n., holotype male, (MZB Cru 4529) **B** *Cherax misolicus* (NMB 956a) **C** *Cherax warsamsonicus* sp. n right first chela, dorsal aspect **D** *Cherax misolicus* right first chela, dorsal aspect.

*Cherax warsamsonicus* sp. n. differs from *C. misolicus* in the following characters: shape of the chelae, (Fig. 8C, D), shape of the rostrum , the presence of setae on the rostrum and in colouration. *Cherax misolicus* has two rostral teeth on each margin of the rostrum while *Cherax warsamsonicus* sp. n. bears 3-4 prominent teeth on each margin. The rostrum of *Cherax misolicus* is rather straight, triangular shaped, while the rostrum of *Cherax warsamonicus* sp. n. is clearly bent outwards at middle part (Fig. 8A, B). *C. warsamsonicus* sp. n. has one prominent corneous spine and three tubercles present at middle part behind cervical groove on lateral sides of carapace while *C. misolicus* has 6–7 small tubercles present there. *Cherax warsamsonicus* sp. n. usually has bluish or dark blue chelae with a white coloured lateral margin and a white patch. Body colour is greenish grey with some pink or red patches on the dorsal carapace right behind the rostral carinae. Pleon is greenish grey with a red to pink pattern dorsally. Legs are usually blue, grey blue or grey. *Cherax misolicus* has light blue chelae, the body is olive green with orange bluish legs and a dark blue pleon with orange on the lateral pleon.

*Cherax warsamsonicus* sp. n. is endemic in the Warsamson River and Warsamson tributaries in West Papua while *C. misolicus* is endemic in creeks and rivers of Misool Island.

###### Etymology.

**Figure 9.**
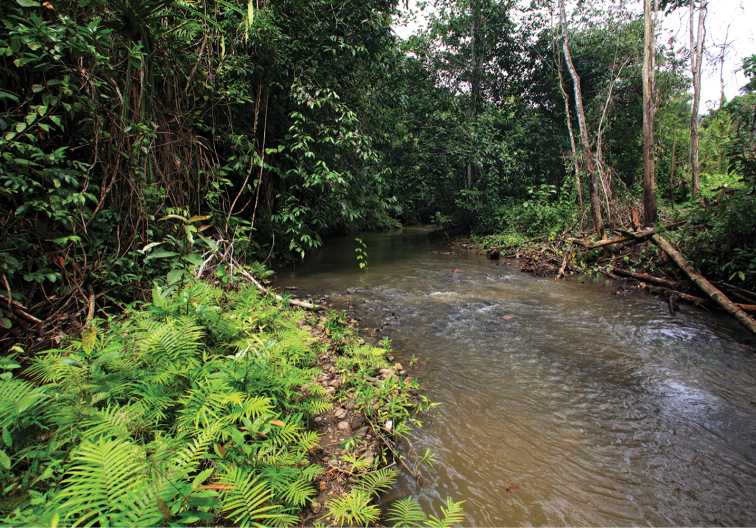
The Bird's Head Peninsula, West Papua, Indonesia with the type locality, Warsamson River, indicated.

*Cherax warsamsonicus* sp. n. is named after the Warsamson River in West Papua where it seems to be endemic (Fig. 9).

###### Ecology.

**Figure 10.**
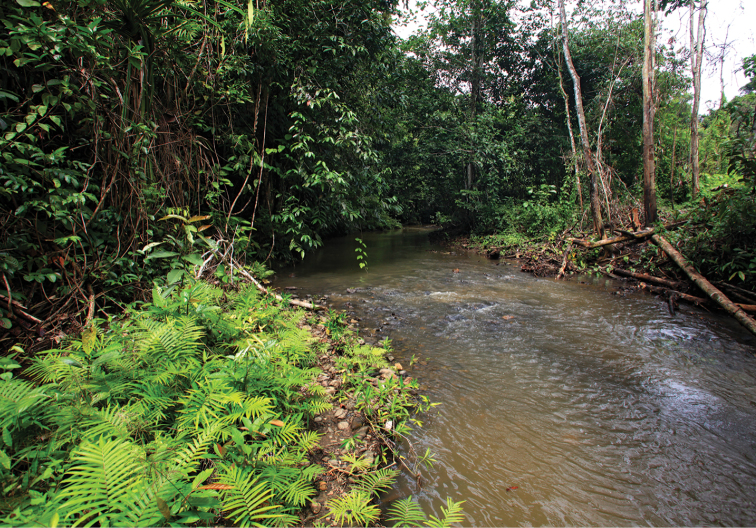
Tributary to the Warsamson River, habitat of the new species.

Known only from the Warsamson River and its tributaries, South Sorong Regency in the central part of the Kepala Burung (Vogelkop) peninsula. The creeks from where these crayfish have been collected are shallow (20–60 cm) with a moderate flow, the water is clear, and have a pH of approximately 6.5. In most of the parts no water plants are present. The substrate of the creek is gravel or sand and soil mostly covered with silt and detritus, stones and larger rocks (Fig. 10). Crayfish hide in short borrows in the riverbank, under lager rocks or in detritus that gathers in slower flowing parts of the creek or river. To improve the knowledge of the distribution of the species more field trips will be necessary.

###### Common name.

The common name of the new species in the pet trade is *Cherax* “irian jaya”, *Cherax* “pink coral”, and sometimes it is sold also as *Cherax pulcher*. Therefore we propose the name Warsamson River Crayfish as a common name for the new species.

## Supplementary Material

XML Treatment for Cherax warsamsonicus
