# Corrigenda: *Cherax warsamsonicus*, a new species of crayfish from the Kepala Burung (Vogelkop) peninsula in West Papua, Indonesia (Crustacea, Decapoda, Parastacidae) ZooKeys 660: 151–167. https://doi.org/10.3897/zookeys.660.11847

**DOI:** 10.3897/zookeys.665.12850

**Published:** 2017-04-04

**Authors:** Christian Lukhaup, Rury Eprilurahman, Thomas von Rintelen

**Affiliations:** 1 Waldstrasse 5a, 66999 Hinterweidenthal, Germany; 2 Animal Systematics Laboratory, Faculty of Biology, Universitas Gadjah Mada Jl. Teknika Selatan, Sekip Utara Yogyakarta 55281, Indonesia; 3 Museum für Naturkunde -Leibniz Institute for Evolution and Biodiversity Science, Invalidenstraße 43, 10115 Berlin, Germany

It has come to our attention that in the work referenced above Table 1 is incomplete. Furthermore, Figure 7 as printed therein is not the final version of that figure.

The correct versions of both Table 1 and Figure 7 are reproduced here below.

**Table 1.** Material studied with GenBank accession numbers.

Species/sample	Location	GenBank acc. nos	
		COI	16S
*Cherax albertisii*	Bensbach River, Papua New Guinea (Queensland Museum)	–	KJ920770
*C. boesemani*	Ajamaru Lake, Papua Barat; 1°17'19.97"S, 132°14'49.14"E; January 23, 2016	KY654084 KY654085	KY654089 KY654090
*C. holthuisi*	Papua Barat	KU821419	KU821433
*C. misolicus*	Misool Island, South of Papua Barat (Leiden Museum)	-	KJ920813
*C. monticola*	Baliem River, Wamena, Papua	KF649851 –	KF649851 KJ920818
*C. paniaicus*	Lake Tage, Papua (Field collection)	KJ950528	KJ920830
*C. peknyi*	Pet Shop	KU821422	KU821435
*C. pulcher*	Hoa Creek (Teminabuan), Papua Barat; 1°28'32.73"S, 132°3'54.94"E; January 23, 2016	KY654083	KY654088
*C. ‚pulcher*‘	Papua Barat (Pet Shop)	KU821424 KU821426	KU821438 KU821437
*C. rhynchotus*	Lake Wicheura, Cape York, Queensland (Queensland Museum)	–	KJ920765
*C. snowden*	Oinsok (Ainsok River Drainage), Papua Barat; 1°11'40.07"S, 131°50'1.14"E; January 24, 2016	KY654082	KY654087
*C. warsamsonicus*	Small tributary to Warsamson River, 0°49'16.62"S, 131°23'3.34"E; January 20, 2016	KY654086	KY654091
*Engaeus strictifrons*	Crawford River, Victoria, Australia	AF493633	AF492812
*Euastacus bispinosus*	Crawford River, Victoria, Australia	AF493634	AF492813

**Figure 7.**
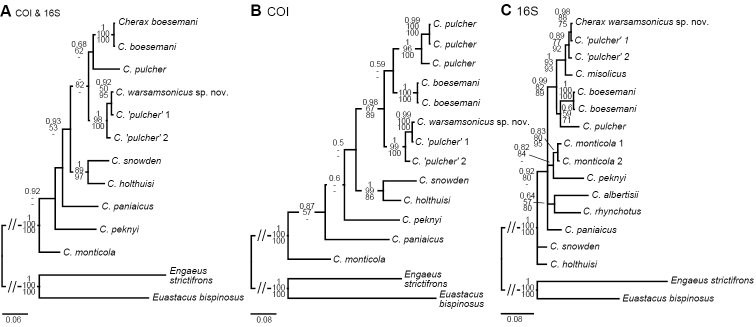
Phylogenetic position of *Cherax warsamsonicus* sp. n. within closely related New Guinean *Cherax* species, reconstructed by BI analyses of two mitochondrial gene fragments. Number on branches show, from top, Bayesian posterior probabilities and ML/MP bootstrap values. The scale bar indicates the substitution rate. See Table 1 for information on the sequenced specimens. **A** Topology based on concatenated COI and 16S dataset **B** Topology based on COI dataset **C** Topology based on 16S dataset.

